# What is “Where”: Physical Reasoning Informs Object Location

**DOI:** 10.1162/opmi_a_00075

**Published:** 2023-05-01

**Authors:** Tal Boger, Tomer Ullman

**Affiliations:** Department of Psychology, Yale University, New Haven, CT, USA; Department of Psychology, Harvard University, Cambridge, MA, USA

**Keywords:** object representation, perception, physical reasoning

## Abstract

A central puzzle the visual system tries to solve is: “what is where?” While a great deal of research attempts to model object recognition (“what”), a comparatively smaller body of work seeks to model object location (“where”), especially in perceiving everyday objects. How do people locate an object, right now, in front of them? In three experiments collecting over 35,000 judgements on stimuli spanning different levels of realism (line drawings, real images, and crude forms), participants clicked “where” an object is, as if pointing to it. We modeled their responses with eight different methods, including both human response-based models (judgements of physical reasoning, spatial memory, free-response “click anywhere” judgements, and judgements of where people would grab the object), and image-based models (uniform distributions over the image, convex hull, saliency map, and medial axis). Physical reasoning was the best predictor of “where,” performing significantly better than even spatial memory and free-response judgements. Our results offer insight into the perception of object locations while also raising interesting questions about the relationship between physical reasoning and visual perception.

## INTRODUCTION

When asked “where is the hammer,” with a hammer right in front of you, where would you point? Initially, this question seems trivial; the hammer is “over there.” Yet, one can give many reasonable answers, each highlighting different properties. Perhaps you would point to the center of the hammer (as it appears to you); or to its handle (the part where you would hold it); or to its metal head (the part that performs the action); or to other locations still. The question “where?” points to a subtle problem in our classic definitions of vision.

Early definitions of vision distilled the complex process of seeing into a simple question: “what is where?” (Marr, 1982). Such definitions served as touchstones for exploring vision in philosophy, cognitive science, and neuroscience, where researchers discovered an apparent split in the visual system between the ventral stream—which models “what”—and the dorsal stream—which models “where” (Schneider, 1969; though more recent work has significantly complicated this initially neat split, as discussed later).

Plenty of research in visual cognition has focused on modeling “what,” and there is an expansive literature about the mechanisms underlying object recognition. While there is also an expansive literature on “where,” by relative comparison it has been less explored than “what,” especially in the perception of everyday objects. Here, we take a step towards exploring the nature of object location by asking: what is “where”?

Much of the existing work on modeling “where” analyzes processes different from simply perceiving objects as they appear in front of us. For example, various work explores the nature of object location via spatial memory (Langlois et al., 2021), ambiguous shapes (Huttenlocher et al., 1991), object parts and scenes (Bar & Ullman, 1996), or eye movements (Vishwanath & Kowler, 2003). These all inform our understanding of object localization in the mind and use methods similar to ours, though in a different context. For example, these works only give hints to where we may point at a hammer if it appeared right in front of us—but do not give a well-defined answer. We expand on these works by testing the nature of perceived object location in simple tasks with everyday objects at differing levels, revealing aspects of object location in our daily lives.

Here, we present three experiments collecting data from over 35,000 judgements in which participants indicate “where” an object is. The experiments use objects covering a wide range of information and realism (such that they generalize to a range of stimuli). We modeled “where” using methods based on previous work, including both human response-based models and image-based models. Across all levels of realism, a model that relies on physical reasoning (perceived center-of-mass)^1^ best predicted “where” an object is. Our results provide novel insights into how we model object locations in perception, and point to a surprising relationship between physical reasoning and visual perception.

## RESULTS

Our three experiments span a range of object realism. In Experiment 1, we used line drawings with no depth and color. In Experiment 2, we used images of real objects with depth and color (but no background). Finally, in Experiment 3, we masked and rotated the line drawings, such that they became unidentifiable crude forms.

**Figure 1.**
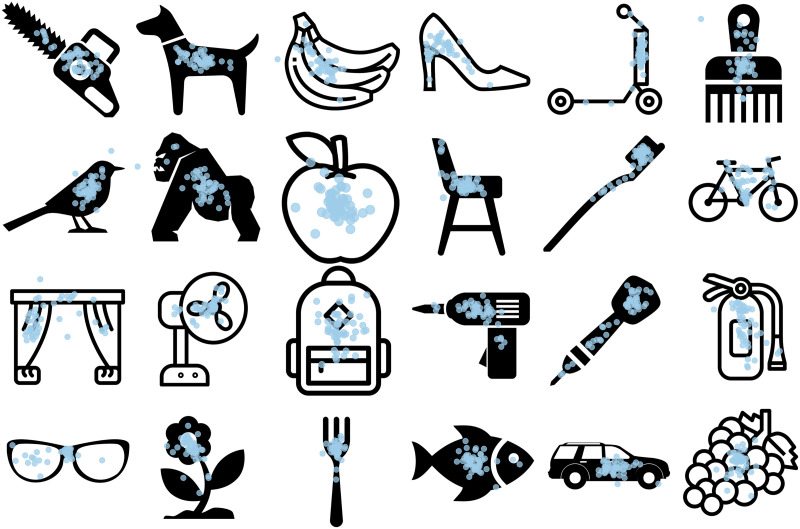
**Example participant data from Experiment 1 (line drawings).** Each blue dot shows a participant’s click in response to the query “where is the [object]?” Plots of “where” data for all our stimuli are accessible on our OSF repository (osf.io/nhj7k). Readers may also try each task for themselves at: tb.perceptionresearch.org/what_is_where.

On each trial, participants clicked “where” each object is, as if pointing it out to another person (Figure 1). Each stimulus set consisted of 50 objects, which included a range of everyday entities, both symmetric and asymmetric items, tools, agents, and more.

We modeled participant responses for “where” using eight different models. The first three models were based on human responses from other tasks, collected separately: (1) center of mass (“click on the object’s center of mass”), (2) spatial memory (“click where the object was” after the object disappeared), and (3) free-response clicks as an attention proxy (“click anywhere on the object”). Participants in each task and experiment were unique and independent. We also considered four image-based models: (4) a uniform distribution across the object, (5) a uniform distribution across the object’s convex hull, (6) the object’s saliency map (as generated by OpenCV fine-grained saliency maps), and (7) medial axis (as generated by scikit-image). The models provide a balance between new proposals specific to this work, and existing models that have been shown to perform well in similar tasks (e.g., medial axis from Firestone & Scholl, 2014). After we tested these broad models of “where,” we pre-registered and analyzed a final, more specific model: (8) human responses on where they would grasp the object to pick it up.

With regards to the center-of-mass model, we emphasize that the true center of mass cannot be accurately recovered, and is also irrelevant even if it could be, as people have no direct access to it. The primary aspect that matters for our analysis based on this model is people’s subjective judgement of the center of mass, and how that relates to the perception of “where.”

**Figure 2.**
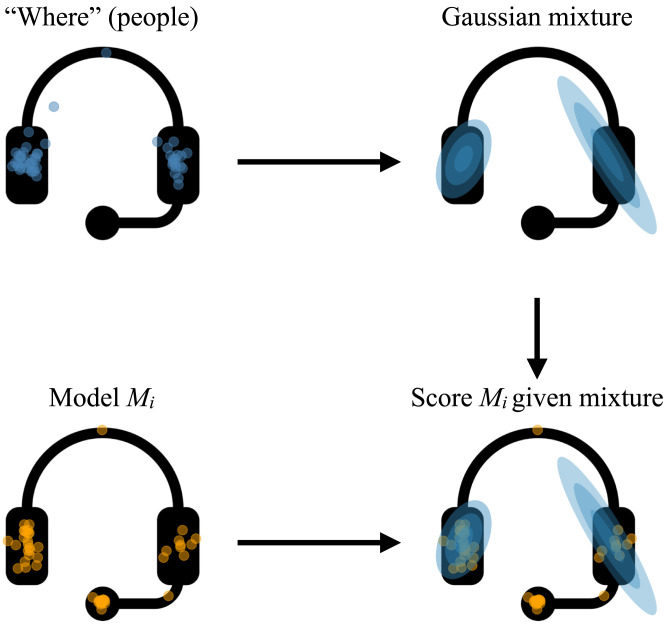
**Schematic illustration of our modeling paradigm, using a line drawing of headphones as an example.** First, we collect participant judgements for “where” an object is. After fitting a Gaussian mixture to this data, we score a proposed model *M*_*i*_ under this Gaussian mixture, using its negative log-likelihood.

To test the performance of our models, we first fit a Gaussian mixture model (GMM) to the “where” data provided by participants for each object, such that we could compare distributions of participant responses (Figure 2). The number of mixtures was chosen via three-fold cross-validation (for between 1 and 5 mixtures). We then calculated the mean negative log-likelihood of our models under this GMM for “where” on each object. Finally, we compared the models using paired Wilcoxon signed-rank tests on the mean negative log-likelihood scores to ask which model best predicted the “where” responses. All analysis plans, choice of models, and experimental designs were pre-registered; materials and data are available at osf.io/nhj7k.

**Figure 3.**
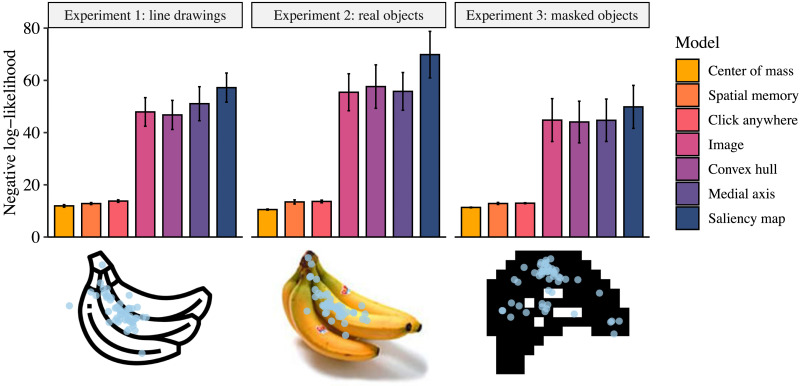
**Mean negative log-likelihood of our initial seven models in each experiment.** In all three experiments, the center-of-mass model performed significantly better than the other models. Under each experiment’s results is a depiction of a banana in that experiment’s form of stimuli, overlaid with “where” data. All analyses and stimuli are available at (osf.io/nhj7k).

In all three experiments, “where” responses were best predicted by center-of-mass judgements, followed by spatial memory judgements and free-response clicks, respectively (Figure 3). The differences between these models was significant: physical reasoning was a significantly better predictor of “where” than spatial memory across experiments (Experiment 1: *p* < 0.01; Experiment 2: *p* < 0.001; Experiment 3: *p* < 0.001). Spatial memory in turn significantly outperformed the free-response model, though the difference was slightly smaller (Experiment 1: *p* = 0.01; Experiment 2: *p* = 0.04; Experiment 3: *p* < 0.01). The various image-based models, while based on previous work and reasonable assumptions, performed poorly by comparison.

Furthermore, our eighth model (where people would grab the object), which we pre-registered and explored after testing our initial seven, performed significantly worse than our initial three human response-based models. In all three experiments, the grasping model performed the worse than the center of mass, spatial memory, and “click anywhere” models (*p* < 0.001 when compared to the center-of-mass model in each experiment).

We estimate ceiling performance as the log likelihood of people’s “where” judgements under its own GMM—another model should not predict the “where” data better than the “where” data itself. In Experiments 1 and 2, the “where” data was significantly more likely under the GMM than the center of mass data (Experiment 1: *p* < 0.01; Experiment 2: *p* < 0.01). However, in Experiment 3, we observe near-ceiling performance for the physical reasoning model, as its likelihood is not distinguishable from the likelihood of the “where” data itself (*p* = 0.43).

Though participants tend to click near the center of objects—perhaps leading to a bias towards the center-of-mass model—this does not explain away our results. First, this bias of clicking near the center of the objects would apply to *all* human-response models, not just the center-of-mass model. Further, had this been the case, then simply predicting responses for “where” by distance from the image centroid would be the best model. However, this was not the case, suggesting that this preference for center of mass goes beyond mere clicking biases.

## DISCUSSION

What is “where”? Our three experiments explore how we judge the location of objects and find that, across a range of object realism, a judgement rooted in physical reasoning (center of mass) is the strongest predictor of perceived object location. We suggest that judgements of object location rely on physical properties. This idea echoes other work in visual perception, neuroscience, and developmental psychology.

Recently, many researchers hypothesized that a mental “simulation engine” underlies much of our intuitive physical reasoning (Battaglia et al., 2013; Fischer et al., 2016). The first step in these mental simulation models assumes we de-render a visual image into a physical scene representation, but this process is not yet formally solved. With a few exceptions (e.g., Boger & Firestone, 2022; Little & Firestone, 2021), the question of how physical reasoning fits into vision remains relatively unexplored.

Our results speak to a different direction in this process; we suggest that physical reasoning and vision rely on each other, rather than one process exclusively relying on the other. This relationship is strong enough that physical reasoning predicts perceived object locations better than seemingly closer processes such as spatial memory and attention. The instructions for the spatial memory judgements (“where was the object”), free-response judgements (“click anywhere on the object”), and “where” judgements (“where is the object”) are all semantically similar, compared to the physical reasoning judgements (“click on the object’s center of mass”). This makes our empirical findings all the more striking.

Beyond intuitive physical reasoning, our work adds a new angle to existing literature on multiple object tracking (MOT) and object-based attention. For example, foundational work in MOT suggests that attention is deployed to objects rather than features, implying that there is a sort of physical “objectness” crucial to vision (Scholl et al., 2001). This tracking ability persists dynamically through some physical events such as occlusion, but not others (such as deletion) (Scholl & Pylyshyn, 1999). Such object-based attention even exists over object representations implicitly created by perceptual completion (Moore et al., 1998). However, much of these results exist over either dynamic objects (i.e., in the case of MOT) or abstract objects. Here, we show the impact of physical “objectness” on the judgements of object location in a simple, static paradigm involving everyday objects.

Our results also have analogs in neuroscience, further aligning with our proposed relationship between physical reasoning and vision. For example, the MT complex—thought to be responsible for motion perception—has been shown to mediate attentive tracking (Culham et al., 1998). A large body of work has taken this classic split between the “what” and “where” streams to include “how,” which modulates how we interact with objects, noting that people can direct accurate motor movements at objects they fail to localize (Goodale & Milner, 1992; Goodale et al., 1991; Kravitz et al., 2011). This matches our results that the “where” of objects may be constrained by their physical behaviors.

At an even more basic level of representation, foundational work in infant cognition shows that physical reasoning may supersede representations of object identity. Even when infants forgot the features of a set of objects, they still expected them to remain physically consistent. For example, infants who fail to notice changes in object shape are surprised to see the object disappear entirely (Kibbe & Leslie, 2011; Zosh & Feigenson, 2012). This provides an even richer demonstration of earlier work showing that infants’ representations of objects conform to more foundational physical properties such as continuity and rigidity (Baillargeon et al., 1985; Spelke et al., 1992; Spelke & Van de Walle, 1993). We suggest that such representations persist across development (as in Kibbe, 2015) in even simpler ways than object tracking or planning, and rather influence our judgements of object locations in simple, static settings.

Though our experiments covered a range of realism, they were still limited to static objects with no background. Physical reasoning in the real world deals with complex scenes and moving objects. Future work may explore how dynamics, relations, and interactions affect judgements of “where.” In everyday life, we do not recognize and locate a hammer as an isolated object, but as a hammer *next to* a cup, *behind* a book, *on* a tiger, and so on (Hafri & Firestone, 2021). These relations require extracting rich physical and visual information that may affect our perception of object location. For example, participating in a causal event such as a collision creates a reliable illusion in the spatial relations of two objects (Scholl & Nakayama, 2004). Future work may explore how “where” changes if, rather than seeing a hammer with no background, we see a hammer supported by a table from below, or supported by a string from above.

Beyond the insights our results provide about physical reasoning and object location, they raise intriguing questions about how “where” relates to “what.” For example, when judging the location of a pineapple, people consistently clicked on its body, as if ignoring the stem on top. What does this mean about the nature of how we perceive the pineapple? Judgements of “where” may reveal a unique way to analyze the perceived essence of an object (Gelman, 2003). Because participants must choose only a single point on the object, they may ask themselves which part of the object most represents its essence. Perhaps, in line with work in infant cognition and MOT, we represent the essence of objects not only by their visual features, but also by their physical properties, in some cases even relying more on the latter than the former.

While our experiments ask each participant for a single judgement, our perception of “where” likely depends on more than just a single point. This representation may instead resemble a “point cloud.” However, we can treat each single-point estimate as a sample drawn from such a cloud distribution, in line with other proposals on sampling-based cognition (Vul et al., 2014). By aggregating judgements across participants, we generate well-powered cumulative distributions of object location, in much the same way that cumulative distributions reveal mathematically defined shape skeletons (Firestone & Scholl, 2014). In this sense, our distributions recapture the potential point-cloud distributions, which turn out to be neither uniform, nor skeletal.^2^

By modeling “where” in a simple and direct setting, we take a step towards understanding how we represent object location. Our results reveal a surprising bidirectional relationship between physical reasoning and visual perception. More broadly, we believe this work suggests a novel avenue for future work on modeling “where” in vision. While it does not fully resolve the question of what is “where,” it suggests where to look.

## FREQUENTLY ASKED QUESTIONS

We thought it would be useful to directly address a few common questions and comments we have received regarding this work. We hope this helps lead to open conversation with readers, and serves as a “theoretical supplement.”

### Surely people represent locations as more than a single dot, something more like an area-cloud?

We agree participant representations of “where” may involve multiple locations on the object, rather than the single location we ask each participant to produce. But, we believe these single point estimates form well-powered point clouds, which together reflect the “where” distribution.

In many ways, giving participants the option to click on the object is the best solution to such a problem. First, such issues exist in other single-choice clicking tasks (e.g., Firestone & Scholl, 2014) which also find mathematically strong distributions. Second, clicking tasks give participants maximum flexibility to represent this single location as best they can, whereas, for example, a forced-choice task adds ambiguity to this point-cloud across participants.

### Wouldn’t any dot on the image be a valid answer? If I point to any part of an image and ask “is this the [object]” the answer should be “yes”

In principle, valid “where” responses would be any location on the surface or edges of the object (though in our experiments people also point to empty areas, such as the middle of a bicycle). In practice, this is not what people do when generating responses. Under the hypothesis that any image part is a valid answer, people would conflate “where” with “any non-background pixel,” and responses should then either form a uniform distribution across the object (a uniform point cloud), or the single-best error-minimizing point sample from that cloud (the image center). We don’t observe either of these. Rather, we see that center of mass is highly predictive of “where” responses.

### Is this about vision? Isn’t this actually about social things, such as communication?

We do not know for sure that our results contain no social component, and it would be interesting if they did. However, we cannot think of a theoretical account at the moment for how social features explain our results, and dictate people’s responses in a way that a hypothetical “social-free” version would not. Put as a question, why would “point an object out to someone” cause participants to produce clicks that match judgements about center of mass (a non-social judgement), but simply locating an object for yourself result in different judgements?

Also, such social or communication components exist (via task demands) in other studies that are taken to be about vision, and cannot be fully removed. For example, (Firestone & Scholl, 2014)—who ran similar tasks to explore shape skeletons in the visual system—ask participants to tap anywhere on a shape. Though there is no language about “pointing it out to someone,” the experiments do require participants to tap the shape on an iPad held by an experimenter, requiring some form of pointing it out to, and communicating with the experimenter.

More broadly, the question about whether this is *actually* about vision in turn raises the question of what vision is, and a classic answer has been “vision is about what is where,” bringing us full circle.

### Have you considered [this other model] instead?

In this work, we analyzed eight different models, which is straining a short paper. We chose these models to form an encompassing package, while trying to not be overbearing, and not claiming to be exhaustive. In the process, it’s quite possible we left out other reasonable models.

We’re happy to explore new models, or additions to the current models. We also encourage proposals for why our existing models work or don’t work. We believe part of the appeal of this work is in spurring new directions. However, we have two suggestions for any new models or proposals.

First, new models or proposals should match the data already at hand, at a basic level. For example, several proposals beyond our set turn out to be equivalent to a uniform distribution or center-of-image model, which does not match the existing data. We considered this above, in the interpretation that “where” ambiguously leads to “any non-background pixel,” and we’ve also come across proposals that people might “minimize the error of a mis-click,” which turn out to be similar.

Second, new models or proposals should be able to generalize in a way that can capture both our broad stimulus set, and visual representations more generally. This is perhaps a main pitfall of our eighth model, that asks participants where they would grab the object. Many objects in our stimulus set (and the world) are not graspable, especially the crude forms we use in Experiment 3.

We invite interested readers to test new models and proposals; all our data and experimental code are available on our OSF repository (osf.io/nhj7k).

### How do we know participants are calculating the center of mass accurately? Why not calculate the true center of mass, instead of relying on people’s judgements?

We would stress that a “true” center of mass cannot be calculated from our images, given that the weight of each object part is unknown. So, there is no way to know such calculations are capturing a ground truth. (Though previous work has shown that people accurately judge an object’s center of mass (Cholewiak et al., 2015).) More importantly, even if we *could* calculate the true center of mass, it would be irrelevant for judgements of “where.” People do not have access to the ground-truth center of mass beyond the mental calculations they perform in the task asking them to estimate the center of mass, which is what we asked them to do.

### How do we know participants are not merely clicking on the center of the object for “where,” and that’s why center of mass is the best model?

As with the above question of additional models, we believe that this concern would need to be first validated by the data. Before performing any analysis, we can see that people are not merely clicking on the object’s center, and rather that the clicks possess a unique distribution which seems to have some structure.

However, this concern can also be tested empirically; if the main reason the center-of-mass model predicts the “where” data well is because of a bias to click towards the center, then a model predicting “where” clicks using the image centroid should perform the strongest. However, this is not the case, as it performs significantly worse than all human response-based model.

Finally, if such a center bias existed in the “where” clicks, it would likely extend to other models. It is especially hard to explain why such a bias would not extend to the “click anywhere” model (and why that model is not the strongest) under this explanation; the instructions for the “click anywhere” and “where” tasks are almost identical, such that a center bias in one *should* extend to the other if it existed. However, this is not what we observe, so we believe this concern is unsubstantiated by our data.

## MATERIALS AND METHODS

### Participants

Each of the three experiments recruited 50 unique participants for each of the tasks and each of the three forms of stimuli (“click where the object is,” “click on the object’s center of mass,” “click where the object was,” “click anywhere on the object,” and “click where you would grab the object to pick it up”; total *n* = 750). All participants were recruited from the online platform Prolific (for a discussion of the reliability of this subject pool, see Peer et al., 2017). Unique participants were used for each condition and experiment such that no participant appeared in more than one model or in both the dependent and independent variables. Participants were excluded if they did not contribute a complete dataset or if they clicked the same location in five consecutive trials.

### Stimuli

Line drawings for Experiment 1 were taken from The Noun Project. Object images for Experiment 2 were taken from a variety of online sources. The kinds of objects in Experiment 2 were the same as those in Experiment 1 (i.e., if Experiment 1 included a line drawing of a gorilla, Experiment 2 included a real image of a gorilla). The masked objects for Experiment 3 were created by applying a random mask to the line drawings, then vertically flipping them to remove any identifying information. The images were randomly padded both vertically and horizontally such that responding in the center of the screen each time would not produce reasonable data. All images were 500 × 500 pixels large in the participant’s web browser.

Note that unique participants are assigned to each condition, where they then see all the stimuli in the given form and answer the given question. In other words, a participant in the Experiment 1 “center of mass” conditions will see 50 line drawings and click on their center of mass; they will not see any images of other types or be told to click according to different instructions. The same set of 50 images are used across all conditions in a given experiment.

### Design and Procedure

Participants saw 50 images in each experiment. The order of the images was randomized. When gathering judgements for “where,” we instructed participants as follows: “Your friend asks: ‘where is the [object]?’. Click on where you would point to.” In the “center of mass” condition, participants were told to “Click on the center of mass of the [object].” Participants in this condition were provided with an additional instruction of what center of mass means (“average position of all the mass in the object”). Though we cannot calculate the “accuracy” of these responses (given that we cannot calculate a true center of mass from images), the responses appear consistent and reasonable (and previous work shows such judgements are fairly consistent; Cholewiak et al., 2015). In the spatial memory condition, the object appeared for 1000 ms, during which time the participant’s mouse was hidden and immovable. The object then disappeared and participants were instructed as follows: “Your friend asks: ‘where was the [object]?’. Click on where you would point to.” In the “click anywhere” condition, participants were told to “Click anywhere you want on the [object].” Finally, the “grasp” condition instructed participants to “Click where you would grab the [object] to pick it up.”

### Data Availability

All data, code, materials, and pre-registrations are available at osf.io/nhj7k. Readers can also do the tasks for themselves at tb.perceptionresearch.org/what_is_where.

## ACKNOWLEDGMENTS

For helpful discussion and comments on previous drafts, we thank Sami Yousif, Chaz Firestone, members of the Harvard CoCoDev lab, and members of the Computation and Language Lab at UC Berkeley.

## FUNDING INFORMATION

TU is supported by NSF Science Technology Center Award CCF-1231216, the DARPA Machine Common Sense program, and the Jacobs Foundation.

## AUTHOR CONTRIBUTIONS

TB carried out the experiments, analyzed the data, and wrote the first draft of the manuscript. TB and TU jointly edited the paper and designed the research.

## Notes

^1^ Note that, while other relevant dimensions for physical reasoning in humans exist beyond center of mass, we use center of mass as a proxy for physical reasoning. Computing center of mass requires some kind of physical reasoning, which previous work has shown to be quite sensitive, or at least inaccurate in consistent ways which still imply a physical computation (Cholewiak et al., 2013, 2015; Firestone & Keil, 2016), making such a proxy reasonable and well-defined.^2^ An additional way to address this point cloud hypothesis would be with a series of object localization tasks that do not rely on clicking, such as a vernier acuity task (for review, see McKee & Westheimer, 1978). Relying on two-alternative forced-choice responses for object positions eliminates aspects of the fine-grained modeling approach we present here. However, it also presents a higher-level, coarser interpretation of object location; future research may seek to use these types of paradigms to further our understanding of object localization. In this work, we stick to clicking-based tasks given their simplicity and prevalence in related work, such as in Firestone and Scholl (2014).
